# Association of high estimated glomerular filtration rate with risk of atrial fibrillation: a nationwide cohort study

**DOI:** 10.3389/fmed.2023.1207778

**Published:** 2023-08-25

**Authors:** Min Kyoung Kang, Hee-Jung Ha, Raon Jung, YunSeo Oh, Dong-Hyeok Kim, Tae-Jin Song

**Affiliations:** ^1^Ewha Womans University Seoul Hospital, Seoul, Republic of Korea; ^2^College of Medicine, Ewha Womans University, Seoul, Republic of Korea

**Keywords:** atrial fibrillation, high estimated glomerular filtration rate, population study, renal function, glomerular hyperfiltration, neurology

## Abstract

**Aim:**

While the relationship between impaired kidney function and atrial fibrillation (AF) is well established, there is limited research exploring the association between elevated estimated glomerular filtration rate (eGFR) and AF development. This study aimed to examine the association between higher-than-normal eGFR and AF risk using a nationwide longitudinal study of the general population in Korea.

**Materials and methods:**

This study utilized the National Health Insurance Service cohort database of Korea, analyzing data from 2,645,042 participants aged 20–79 years who underwent health examinations between 2010 and 2011. Participants with a history of end-stage renal disease, renal transplantation, and AF prior to the index date were excluded. Renal function was assessed using eGFR levels, calculated with the Chronic Kidney Disease Epidemiology Collaboration (CKD-EPI) equation. Baseline characteristics were gathered through questionnaires, while comorbidities and AF occurrence outcomes were identified and validated using diagnostic codes and medication histories. The study employed Kaplan–Meier survival curves and Cox proportional hazard models to evaluate the association between eGFR and AF occurrence.

**Results:**

The mean age of subjects was 48.82 ± 10.08 years. Over a median follow-up of 9.58 years, 27,469 (1.04%) AF cases were identified. The risk for AF increased in the higher-than-normal decile, as demonstrated by Kaplan–Meier survival curves (*p* < 0.001). The eGFR <30 mL/min/1.73 m^2^ group was associated with an increased risk of AF [hazard ratio (HR): 1.22, 95% confidence interval (CI) (1.01, 1.46), *p* = 0.039], while the eGFR >120 mL/min/1.73 m^2^ group was associated with a decreased risk of AF [HR: 0.88, 95% CI (0.78, 0.98), *p* = 0.045]. Compared to the 5th decile, the 1st [HR: 1.08, 95% CI (1.03, 1.13), *p* = 0.010] eGFR decile was significantly associated with an increased risk of AF, while the 10th [HR: 0.77, 95% CI (0.70, 0.85), *p* < 0.001] eGFR decile was significantly associated with a reduced risk of AF.

**Conclusion:**

The study revealed that individuals with eGFR>120 mL/min/1.73 m^2^ or those falling within eGFR 10th decile (>113.41 mL/min/1.73 m^2^) demonstrated an inverse association linked to a reduced risk of AF. Our study suggests that general population with higher-than-normal eGFR levels may have a lower risk of developing AF.

## Introduction

Renal function has been linked to cardiovascular diseases, including atrial fibrillation (AF), stroke, myocardial infarction, and heart failure. Chronic kidney disease, often characterized by low estimated glomerular filtration rate (eGFR), is strongly associated with the risk of these cardiovascular diseases (1, 2). In addition to low eGFR, abnormally high eGFR has also been linked to various health conditions. While high eGFR is generally considered a normal physiological state, it can also indicate underlying adverse renal conditions, such as the onset of glomerular damage in hypertensive patients or diabetic nephropathy (3). High eGFR is also closely associated with hypertension, prediabetes, and obesity, all of which may contribute to cardiovascular events (4).

Atrial fibrillation is a common arrhythmia associated with an increased risk of stroke, systemic thromboembolism, and mortality (5–8). As the global population ages and concomitant vascular risk factors rise, the worldwide burden of AF continues to grow (9–12). The relationship between diminished kidney function and AF is well-established. Chronic kidney disease and AF share common vascular risk factors, such as hypertension and diabetes (13). Reduced kidney function leads to endothelial dysfunction, and these pathological conditions heighten the risk of AF (14).

Given that high eGFR typically reflects favorable renal function, it is plausible that a population with high eGFR presents a low risk of developing AF. However, since high eGFR might indicate early changes related to renal disease, the risk of AF could be inversely high. Limited studies have examined the relationship between high eGFR and the development of AF. This study aims to investigate the association between higher-than-normal eGFR and the risk of AF in a nationwide longitudinal study of the general population in Korea.

## Materials and methods

### Data source

The data for this study were obtained from the Korean National Health Insurance Service-Health Screening cohort database (NHIS-HEALS). The NHIS is a government initiative that provides health insurance for 97% of the country’s population, with the remaining population covered by the Medical Aid program, which is also supervised by the NHIS (15–17). To facilitate early disease detection and prevention, annual standardized health screenings are recommended for NHIS enrollees. The NHIS-HEALS database encompasses information on participants’ demographic characteristics, socioeconomic status, health screening outcomes, registered diagnoses, and treatment details. The health screening procedure entails measuring physical parameters such as height, weight, and blood pressure, conducting laboratory tests, and surveying lifestyle habits (18–23).

### Study population

**Figure 1:**
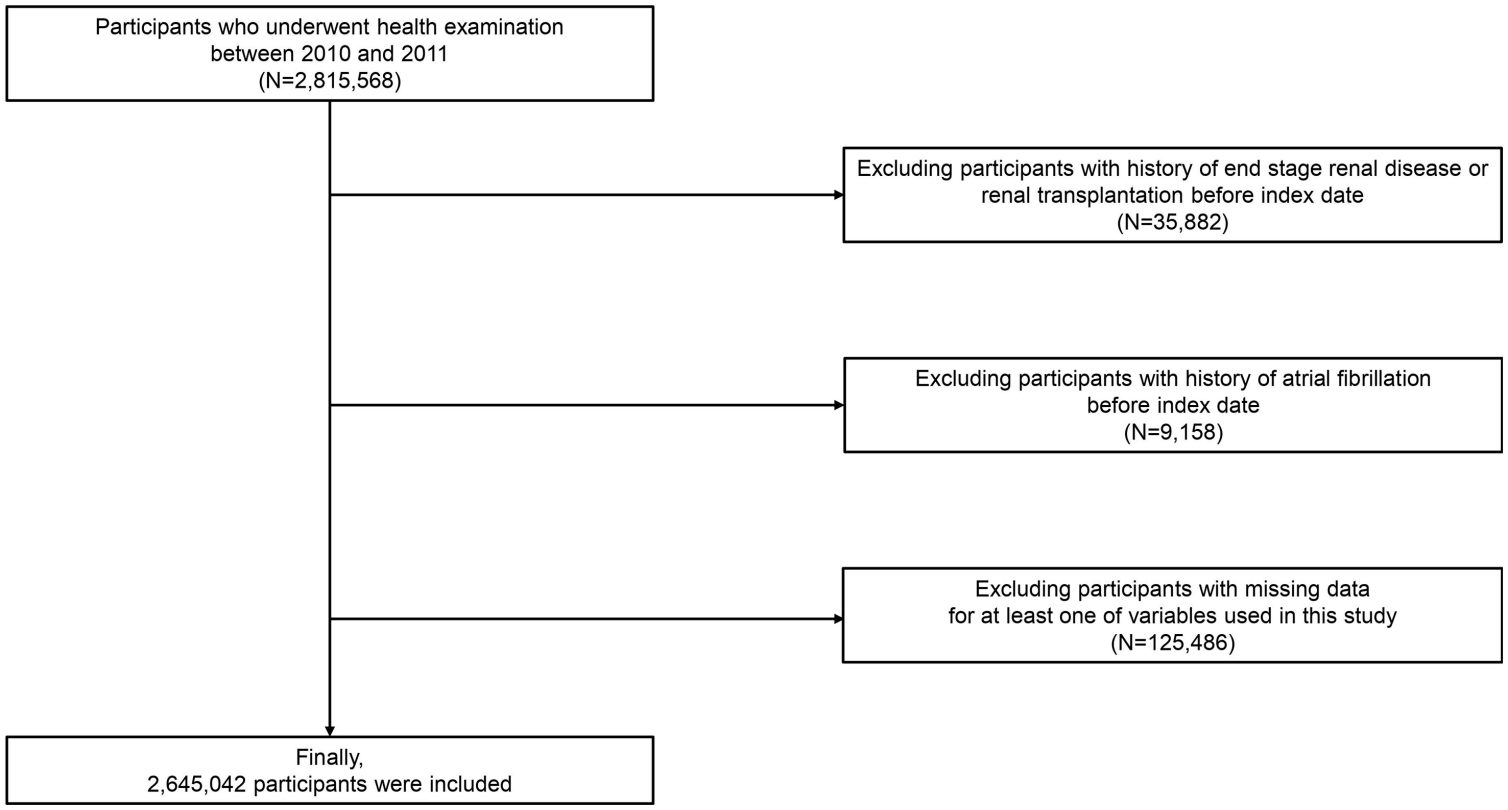
Flowchart depicting the selection process for study participants.

Our NHIS-HEALS cohort comprised 2,815,568 individuals aged 20 to 79 who underwent health screenings between 2010 and 2011 (dataset number: NHIS-2022-01-313) (16, 24, 25). We excluded participants with a history of end-stage renal disease or renal transplantation prior to the index date (*n* = 35,882). Subsequently, we excluded participants (*n* = 9,158) with a history of AF before the index date and participants (*n* = 125,486) missing data for at least one variable. Ultimately, 2,645,042 participants were included in the analysis (Figure 1).

### Definitions and variables

The date of each participant’s health assessment was designated as the index date for the commencement of follow-up. The eGFR was calculated using the serum creatinine (Scr) level obtained during hospital visits and the Chronic Kidney Disease Epidemiology Collaboration (CKD-EPI) equations (Supplementary Methods) (26).

Baseline characteristics were assessed on the index date, encompassing age, sex, body mass index, waist circumference, and household income. Information on smoking, alcohol consumption, and physical activity was gathered using questionnaires. Smoking status was categorized as non-smoker, former smoker, or current smoker. Alcohol consumption and regular physical activity were recorded as frequency per week. Proteinuria was deemed present if the dipstick urine test result was ≥ +1. Comorbidities, including hypertension, diabetes mellitus, dyslipidemia, heart failure, myocardial infarction, valvular heart disease, cardiomyopathy, congenital heart disease, and hyperthyroidism were identified using specific criteria between January 2009 and the index date (Supplementary Methods). Diagnostic codes were classified based on the International Classification of Diseases (ICD)-10, following previous studies (27–32). The primary outcome was the incidence of AF (ICD-10 code: I48), In this study, AF was defined by at least two out-patient hospital visits or one admission with the diagnostic code I48 (33). This diagnostic code was validated via electrocardiogram review, demonstrating a predictive value of 94.1% (34, 35).

### Statistical analysis

The study presented data in two formats: mean ± standard deviation, or as number and percentage. The relationship between eGFR and AF occurrence was examined by categorizing participants into deciles of eGFR or into ranges of eGFR (<30, 30–60, 60–90, 90–120, and > 120 mL/min/1.73 m^2^), with the 5th decile and the range of 60–90 mL/min/1.73 m^2^ serving as the reference group. We employed two different grouping methodologies, which allowed us to explore the data comprehensively, identify potential trends, and interpret the results as more clinically relevant. Kaplan–Meier survival curves were employed to analyze this relationship, while log-rank tests were utilized to compare differences between eGFR deciles and ranges. Cox proportional hazard models were used to calculate hazard ratios (HR) and 95% confidence intervals (CI) for the relationship between eGFR and AF occurrence. Multivariable regression models were applied to account for potential confounding variables, including age, sex, body mass index, waist circumference, income levels, smoking, alcohol consumption, regular physical activity, proteinuria, hypertension, diabetes mellitus, dyslipidemia, and Charlson comorbidity index. Subgroup analyses were conducted based on these confounding variables. For sensitivity analysis, further evaluation was performed using eGFR levels according to the Modification of Diet in Renal Disease (MDRD) study equation (36). Statistical analyses were conducted using SAS software (version 9.2, SAS Institute, Cary, NC, United States), with a *p*-value of less than 0.05 considered significant.

### Ethical approval statement

The Institutional Review Board of Ewha Womans University College of Medicine approved this study and provided a consent waiver (Institutional Review Board approval number: SEUMC 2022–02-018), as the NHIS permitted unrestricted access to anonymized data for research purposes.

## Results

The average age of the participants was 48.82 ± 10.08 years, with males comprising 51.4% of the population. The prevalence of hypertension, diabetes mellitus, and current smokers was 24.9%, 10.6%, and 19.8%, respectively. With respect to the eGFR deciles, 9.6% of participants fell into the 5th group (reference), exhibiting an eGFR of 86.05–90.49 mL/min/1.73m^2^. Regarding eGFR ranges, the proportions of subjects with eGFR levels <30, 30–60, 60–90 (reference), 90–120, and > 120 mL/min/1.73m^2^ were 0.1%, 4.2%, 44.8%, 46.7%, and 4.1%, respectively (Table 1). In our study, we observed that the glomerular filtration group >120 mL/min/1.73m^2^ tended to be younger in age, with a lower average BMI and smaller waist circumferences compared to other groups (Supplementary Table S1). Additionally, we found a lower prevalence of hypertension, diabetes mellitus, dyslipidemia, and heart failure among individuals in the glomerular filtration group >120 mL/min/1.73m^2^ group (Supplementary Table S1).

**Table 1:** Baseline characteristics of participants.

Variables	Total (*N* = 2,645,042)
Sex	
Male	1,358,515 (51.4)
Female	1,286,527 (48.6)
Age, years	48.82 ± 10.08
Body mass index (kg/m^2^)	23.76 ± 3.27
Waist circumference (cm)	80.30 ± 9.37
Household income	
Q1, lowest	717,496 (27.1)
Q2	922,008 (34.9)
Q3	664,499 (25.1)
Q4, highest	341,039 (12.9)
Smoking status	
Never	1,629,714 (61.6)
Former	388,337 (14.7)
Current	626,991 (23.7)
Alcohol consumption (days/week)	
None	1,411,227 (53.4)
1–4	1,127,425 (42.6)
≥5	106,390 (4.0)
Regular physical activity (days/week)	
None	1,621,142 (61.3)
1–4	869,808 (32.9)
≥5	154,092 (5.8)
Proteinuria	
Negative (−)	2,524,572 (95.5)
Positive (+)	120,470 (4.6)
Comorbidities	
Hypertension	659,503 (24.9)
Diabetes mellitus	279,725 (10.6)
Dyslipidemia	523,785 (19.8)
Heart failure	33,429 (1.3)
Myocardial infarction	7,813 (0.3)
Valvular heart disease	5,819 (0.2)
Cardiomyopathy	1,902 (0.1)
Congenital heart disease	833 (0.0)
Hyperthyroidism	29,648 (1.1)
Charlson comorbidity index	
0	2,096,358 (79.3)
1	402,157 (15.2)
≥2	146,527 (5.5)
eGFR (decile), mL/min/1.73 m^2^	
1st (<67.51)	263,451 (10.0)
2nd (67.51–75.08)	266,010 (10.1)
3rd (75.08–80.69)	265,650 (10.0)
4th (80.69–86.05)	271,300 (10.3)
5th (86.05–90.49)	254,182 (9.6)
6th (90.49–95.50)	262,462 (9.9)
7th (95.50–100.55)	271,764 (10.3)
8th (100.55–105.63)	261,435 (9.9)
9th (105.63–113.41)	264,354 (10.0)
10th (≥113.41)	264,434 (10.0)
eGFR (range), mL/min/1.73 m^2^	
<30	2,774 (0.1)
30–60	112,121 (4.2)
60–90	1,185,354 (44.8)
90–120	1,235,776 (46.7)
>120	109,017 (4.1)

Data are expressed as the mean ± standard deviation, or number (percentage). Q, quartile; eGFR, estimated glomerular filtration rate.

**Figure 2:**
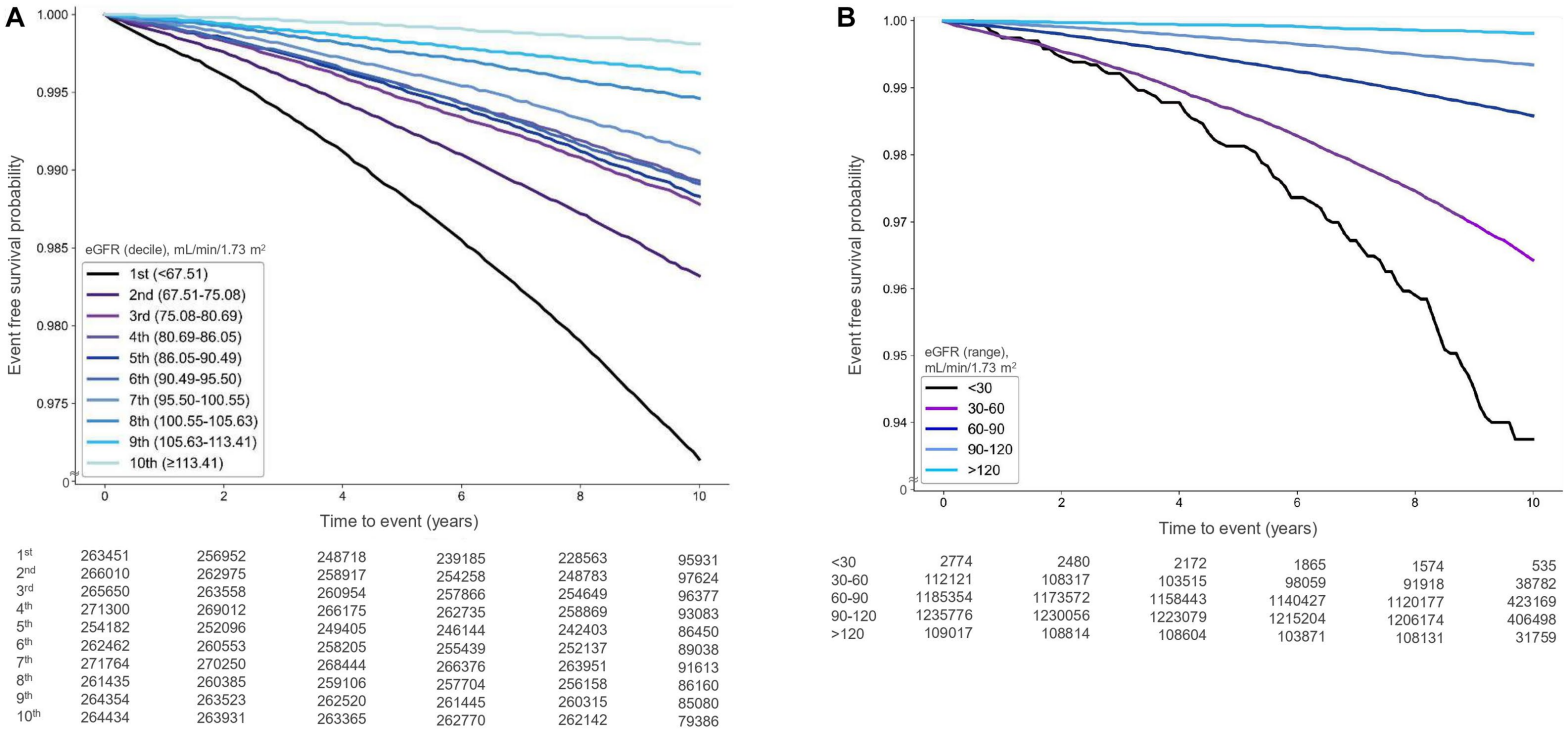
Kaplan–Meier survival curves illustrating the relationship between estimated glomerular filtration rate (eGFR) and atrial fibrillation occurrence [**(A)** deciles, **(B)** ranges].

During a median follow-up of 9.58 years (interquartile range: 9.19–10.11 years), 27,469 cases of AF (1.04%) were documented. Kaplan–Meier survival analysis indicated that the risk of AF was significantly associated with higher-than-normal eGFR deciles (*p* < 0.001) and higher-than-normal eGFR levels (*p* < 0.001) (Figures 2A,B).

**Figure 3:**
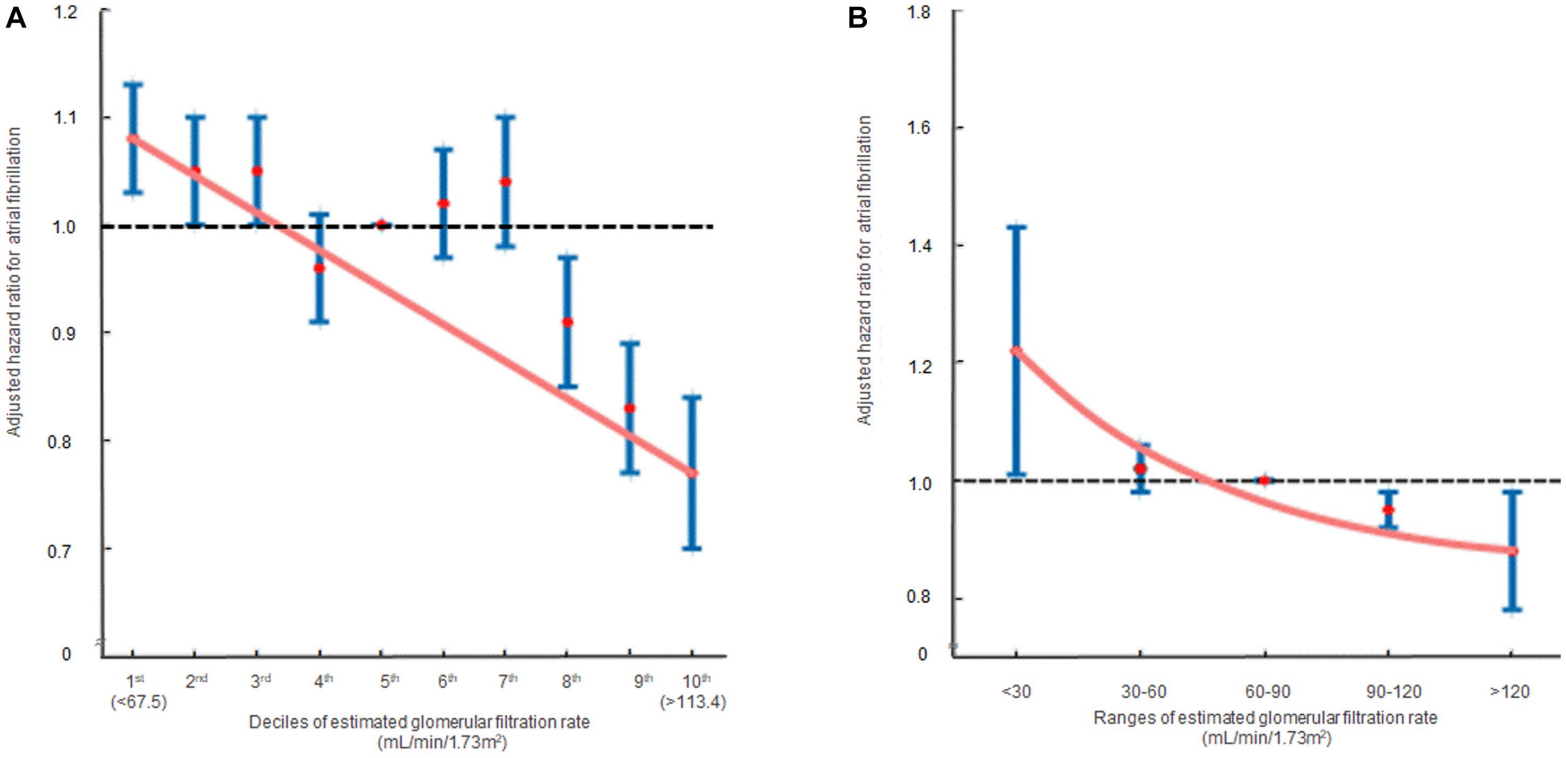
The hazard ratios representing the association between renal function and atrial fibrillation occurrence are displayed in two formats: **(A)** decile groups and **(B)** range groups. The solid blue line shows the multivariate adjusted hazard ratios with 95% confidence intervals for each group, while the dashed lines indicate a hazard ratio of 1. The red line represents the restricted cubic spline curves. Hazard ratios were calculated using the multivariable Cox model shown in Table 2.

In the multivariate analysis, compared to the 5th decile, the 1st [HR: 1.08, 95% CI (1.03, 1.13), *p =* 0.010] and 2nd [HR: 1.05, 95% CI (1.00, 1.10), *p* = 0.034] eGFR deciles were significantly associated with an increased risk of AF. Conversely, the 8th [HR: 0.91, 95% CI (0.85, 0.97), *p* = 0.006], 9th [HR: 0.83, 95% CI (0.77, 0.89), *p* < 0.001], and 10th [HR: 0.77, 95% CI (0.70, 0.85), *p* < 0.001] eGFR deciles were significantly associated with a reduced risk of AF (Table 2; Supplementary Table S2). In terms of eGFR ranges, eGFR <30 mL/min/1.73m^2^ [HR: 1.22, 95% CI (1.01, 1.46), *p* = 0.039] was associated with an elevated risk of AF, whereas eGFR >120 mL/min/1.73m^2^ [HR: 0.88, 95% CI (0.78, 0.98), *p* = 0.045] was associated with a decreased risk of AF (Table 2; Supplementary Table S3). The hazard ratio plot demonstrated a decline in the hazard ratio for AF as GFR increased, both in decile groups and ranges (Figure 3).

**Table 2:** Relationship between renal function and atrial fibrillation incidence.

	Number of participants	Number of events	Event rate (%) (95% CI)	Person-years	Incidence rate (per 1,000 person-years)	Adjusted HR (95% CI)	*p*-value
eGFR (decile), mL/min/1.73 m^2^							
1st (< 67.51)	263,451	6,838	2.60 (2.53, 2.66)	2,403,195.33	2.85	1.08 (1.03, 1.13)	0.010
2nd (67.51–75.08)	266,010	4,236	1.59 (1.54, 1.64)	2,512,757.54	1.69	1.05 (1.00, 1.10)	0.034
3rd (75.08–80.69)	265,650	3,065	1.15 (1.11, 1.19)	2,532,633.50	1.21	1.05 (1.00, 1.11)	0.056
4th (80.69–86.05)	271,300	2,752	1.01 (0.98, 1.05)	2,575,152.64	1.07	0.96 (0.91, 1.01)	0.130
5th (86.05–90.49)	254,182	2,815	1.11 (1.07, 1.15)	2,413,161.20	1.17	1 (Reference)	
6th (90.49–95.50)	262,462	2,721	1.04 (1.00, 1.08)	2,498,383.35	1.09	1.02 (0.97, 1.08)	0.443
7th (95.50–100.55)	271,764	2,279	0.84 (0.80, 0.87)	2,600,312.54	0.88	1.04 (0.98, 1.10)	0.216
8th (100.55–105.63)	261,435	1,352	0.52 (0.49, 0.54)	2,506,681.16	0.54	0.91 (0.85, 0.97)	0.006
9th (105.63–113.41)	264,354	944	0.36 (0.33, 0.38)	2,531,735.26	0.37	0.83 (0.77, 0.89)	<0.001
10th (≥ 113.41)	264,434	467	0.18 (0.16, 0.19)	2,531,292.14	0.18	0.77 (0.70, 0.85)	<0.001
eGFR(range), mL/min/1.73 m^2^							
< 30	2,774	116	4.18 (3.42, 4.94)	19,998.23	5.80	1.22 (1.01, 1.46)	0.039
30–60	112,121	3,494	3.12 (3.01, 3.22)	994,408.94	3.51	1.02 (0.98, 1.06)	0.308
60–90	1,185,354	15,896	1.34 (1.32, 1.36)	11,227,881.41	1.42	1 (Reference)	
90–120	1,235,776	7,770	0.63 (0.61, 0.64)	11,820,400.43	0.66	0.95 (0.92, 0.98)	<0.001
> 120	109,017	193	0.18 (0.15, 0.20)	1,042,615.65	0.19	0.88 (0.78, 0.98)	0.045

The multivariable model was adjusted for sex, age, body mass index, waist circumference, income levels, smoking, alcohol consumption, regular physical activity, proteinuria, hypertension, diabetes mellitus, dyslipidemia, heart failure, myocardial infarction, valvular heart disease, cardiomyopathy, congenital heart disease, hyperthyroidism, and Charlson comorbidity index. CI, confidence interval; HR, hazard ratio; eGFR, estimated glomerular filtration rate.

Subgroup analysis revealed no significant interactions between eGFR ranges and AF occurrence based on sex, body mass index, smoking status, alcohol consumption, regular physical activity, proteinuria, and comorbidities including hypertension, diabetes mellitus, dyslipidemia, heart failure, and myocardial infarction, with the exception of age (dichotomized at the median value of 50 years) (Supplementary Table S4). Sensitivity analysis consistently showed the association of eGFR deciles and ranges with AF occurrence, even when eGFR levels were assessed using the MDRD method (Supplementary Table S5).

## Discussion

Our study revealed that individuals with eGFR>120 mL/min/1.73m^2^ or those falling within eGFR 10th decile (>113.41 mL/min/1.73m^2^) demonstrated an inverse association linked to a reduced risk of AF, which remains significant in individuals presenting with proteinuria, obesity, hypertension, and diabetes mellitus.

Numerous studies have investigated the relationship between eGFR and AF. A community-based observational cohort study conducted in Japan, involving approximately 240,000 individuals, revealed that the hazard ratio for AF occurrence increased proportionally with the decline in renal function over a mean follow-up period of 5.9 years (37). A proportional relationship between the extent of renal function impairment and a higher occurrence of AF was also discovered (38). However, evidence is scarce regarding whether the risk of AF elevates with high eGFR levels. Contrarily, previous studies have reported “U” or “J”-shaped relationships between eGFR and cardiovascular risk or mortality, suggesting that both low and high eGFR levels are associated with an increased mortality risk. When compared to normal eGFR levels, a relatively high eGFR was linked to an increased risk of cardiovascular diseases and mortality. In a prior study of the Chronic Kidney Disease Epidemiology Collaboration dataset, participants with a high eGFR, defined as 95th percentiles of the age- and sex-specific eGFR quintile [HR 1.5, 95% CI (1.2–2.1)] exhibited a significantly heightened risk of cardiovascular events compared to those with normal eGFR (39). In a prospective cohort study comprising 16,958 participants without clinically evident vascular disease, an eGFR >90 mL/min/1.73m^2^ was associated with coronary heart disease and nonvascular mortality (40). Furthermore, a retrospective study of roughly 43,500 individuals from a general population health screening cohort with a mean observation period of 12.4 years identified a correlation between high eGFR, defined as GFR.95th percentile after adjustment for age, sex, muscle mass, and history of diabetes and/or hypertension medication, and increased risk of all-cause mortality (41). In summary, both low and high eGFR levels may be associated with cardiovascular events, including mortality.

Generally, although a high eGFR is frequently deemed favorable due to its indication of good kidney function, it can serve as a marker for underlying health conditions such as hypertension, diabetes, and obesity. As these conditions worsen, the risk of cardiovascular events and mortality increases. Moreover, high eGFR may be associated with dysfunction of the renin-angiotensin system, low-grade vascular or systemic inflammation, endothelial dysfunction, and increased arterial stiffness. These factors represent the primary mechanisms contributing to the development of cardiovascular disease and increased mortality risk (39, 42, 43). However, in our results, the association between high eGFR and increased risk of AF was not substantiated. In other words, high eGFR exhibited a lower risk of AF development, as shown in eGFR>120 mL/min/1.73 m^2^ or eGFR 10th decile (>113.41 mL/min/1.73 m^2^) groups. Although pinpointing an exact mechanism for this relationship is challenging, several hypotheses could explain these findings. Our investigation focused on the risk of AF as the outcome, not the cardiovascular outcome. While impaired kidney function represented by low eGFR levels is associated with both cardiovascular disease and AF development, the association of high eGFR with cardiovascular disease or AF could differ. In essence, high eGFR might display a protective effect against AF risk with exceptionally good renal function in its pure form. Kidney function is intimately connected to the regulation of electrolytes and fluid balance in the body, and disturbances in these processes may contribute to AF development (44). The state of renal hyperfiltration sustains the overall nephron function by utilizing the renal reservoir, thereby maintaining electrolyte and fluid balance, which might signify a low-risk stage before AF occurrence. Additionally, even if high eGFR is presumed to be a preclinical exacerbating factor for the risk of various vascular diseases, it is possible that high eGFR exerts a preconditioning effect on AF occurrence, akin to the ischemic preconditioning effect for arrhythmia (45). Furthermore, since our study included only Asian participants, the results may not be generalizable to Western populations. Additionally, our dataset, which included a nationwide general population, differed from previous studies that involved stroke, diabetes mellitus, and atherosclerosis patients (4). Further research is required to validate these hypotheses.

Our research has limitations that should be acknowledged. First, there may have been a potential for ethnic selection bias, which could limit the applicability of our results to other populations. Additional research on diverse races and ethnicities is warranted. Second, we only assessed cross-sectional eGFR and did not perform cystatin C measurements, which were not included in the NHIS dataset. Additionally, there is a possibility of an overestimation of renal function in some individuals within the group with eGFR >90 mL/min/1.73 m^2^, which could potentially impact the validity of the findings. Lastly, while our study was a longitudinal, nationwide investigation, its retrospective nature might impede the establishment of a causal relationship.

## Conclusion

In summary, this study revealed that individuals with eGFR>120 mL/min/1.73m^2^ or those falling within eGFR 10th decile (>113.41 mL/min/1.73m^2^) demonstrated an inverse association linked to a reduced risk of AF. The anticipated “U”- or “J”-shaped phenomenon was not observed in the relationship between renal function and AF occurrence. Our study suggests that the general population with higher-than-normal eGFR levels may have a lower risk of developing AF.

## Data availability statement

The data analyzed in this study is subject to the following licenses/restrictions: the data used in this study are available in the National Health Insurance Service-National Health Screening Cohort (NHIS-HEALS) database, but restrictions apply to the public availability of these data used under license for the current study. Requests for access to the NHIS data can be made through the National Health Insurance Sharing Service homepage (http://nhiss.nhis.or.kr/bd/ab/bdaba021eng.do). For access to the database, a completed application form, research proposal, and application for approval from the Institutional Review Board should be submitted to the inquiry committee of research support in the NHIS for review. Requests to access these datasets should be directed to http://nhiss.nhis.or.kr/bd/ab/bdaba021eng.do.

## Ethics statement

The studies involving humans were approved by the Institutional Review Board of Ewha Womans University College of Medicine (Institutional Review Board approval number: SEUMC 2022-02-018). The studies were conducted in accordance with the local legislation and institutional requirements. The ethics committee/institutional review board waived the requirement of written informed consent for participation from the participants or the participants’ legal guardians/next of kin because the NHIS permitted unrestricted access to anonymized data for research purposes.

## Author contributions

MK, D-HK, and T-JS contributed to data interpretation and manuscript drafting. MK, H-JH, RJ, YO, D-HK, and T-JS participated in data analysis and interpretation. T-JS contributed to the conception, design, data acquisition, interpretation, and critical revision of the manuscript. All authors contributed to the article and approved the submitted version.

## Funding

This project was supported by a grant from the Basic Science Research Program through the National Research Foundation of Korea funded by the Ministry of Education (2021R1F1A1048113 to T-JS,). This work was supported by the Institute of Information & Communications Technology Planning & Evaluation (IITP) grant funded by the Korean government (MSIT) (2022-0-00621 to T-JS, Development of artificial intelligence technology that provides dialog-based multi-modal explainability). This research was supported by a grant from the Korea Health Technology R&D Project through the Korea Health Industry Development Institute (KHIDI), funded by the Ministry of Health & Welfare, Republic of Korea (grant numbers: HI22C073600, RS-2023-00262087 to T-JS). The funding source had no role in the design, conduct, or reporting of this study.

## Conflict of interest

The authors declare that the research was conducted in the absence of any commercial or financial relationships that could be construed as a potential conflict of interest.

## Publisher’s note

All claims expressed in this article are solely those of the authors and do not necessarily represent those of their affiliated organizations, or those of the publisher, the editors and the reviewers. Any product that may be evaluated in this article, or claim that may be made by its manufacturer, is not guaranteed or endorsed by the publisher.
