# Supplementary material for: Association of high estimated glomerular filtration rate with risk of atrial fibrillation: a nationwide cohort study
Source: Front Med (Lausanne). 2023 Aug 25;10:1207778. doi: 10.3389/fmed.2023.1207778 (PMC10483117; doi:10.3389/fmed.2023.1207778)
Supplement: Supplementary file 2 [file Data_Sheet_1.docx]

**Title: Association of high estimated glomerular filtration rate with risk of atrial fibrillation: A nationwide cohort study**

**Running title**: Association of high eGFR with AF

**Authors**

Min Kyoung Kang^1#^, Hee-Jung Ha^1#^, Raon Jung^2^, YunSeo Oh^2^, Dong-Hyeok Kim^3^, Tae-Jin Song^1*^

^1^Department of Neurology, Seoul Hospital, Ewha Womans University College of Medicine, Seoul, Republic of Korea

^2^Ewha Womans University College of Medicine, Seoul, Republic of Korea

^3^Department of Cardiology, Seoul Hospital, Ewha Womans University College of Medicine, Seoul, Republic of Korea

^#^Both authors contributed equally as first authors.

**Corresponding author:**

Tae-Jin Song, MD, PhD

Department of Neurology, Seoul Hospital, Ewha Womans University College of Medicine, 260, Gonghang-daero, Gangseo-gu, 07804, Seoul, Republic of Korea

Tel: +82-2-6986-1672, Fax: +82-2-6986-7000; E-mail: knstar@ewha.ac.kr

ORCID ID: 0000-0002-9937-762XSupplementary Table 2. Association factors of renal function (deciles of estimated glomerular filtration) with the occurrence of atrial fibrillation

| Variable | Crude HR (95% CI) | p-value | Adjusted HR (95% CI) | p-value |
| --- | --- | --- | --- | --- |
| Sex |  |  |  |  |
| Male | 1(reference) |  | 1(reference) |  |
| Female | 0.67 (0.65, 0.68) | <.001 | 0.56  (0.55, 0.58) | <.001 |
| Age, years | 1.08 (1.08, 1.08) | <.001 | 1.07  (1.07, 1.07) | <.001 |
| Household income |  |  |  |  |
| Q1, lowest | 1(reference) |  | 1(reference) |  |
| Q2 | 0.92 (0.89, 0.95) | <.001 | 0.98  (0.95, 1.02) | 0.338 |
| Q3 | 1.19 (1.15, 1.23) | <.001 | 0.995  (0.96, 1.03) | 0.785 |
| Q4, highest | 1.58 (1.53, 1.64) | <.001 | 1.04  (1.00, 1.08) | 0.053 |
| Smoking status |  |  |  |  |
| Never | 1(reference) |  | 1(reference) |  |
| Former | 1.59 (1.54, 1.64) | <.001 | 1.06  (1.02, 1.09) | 0.003 |
| Current | 0.78 (0.75, 0.80) | <.001 | 0.92  (0.88, 0.95) | <.001 |
| Alcohol consumption (days/week) |  |  |  |  |
| None | 1(reference) |  | 1(reference) |  |
| 1-4 | 0.70 (0.68, 0.72) | <.001 | 1.10  (1.07, 1.13) | <.001 |
| ≥ 5 | 1.68 (1.61, 1.76) | <.001 | 1.26  (1.20, 1.32) | <.001 |
| Regular physical activity (days/week) |  |  |  |  |
| None | 1(reference) |  | 1(reference) |  |
| 1-4 | 0.76 (0.74, 0.78) | <.001 | 0.97  (0.94, 0.99) | 0.017 |
| ≥ 5 | 1.27 (1.21, 1.32) | <.001 | 1.00  (0.96, 1.05) | 0.919 |
| Body mass index (kg/m2) | 1.07 (1.07, 1.08) | <.001 | 1.04  (1.03, 1.04) | <.001 |
| Waist circumference (cm) | 1.01 (1.01, 1.01) | <.001 | 1.01  (1.01, 1.01) | <.001 |
| Proteinuria |  |  |  |  |
| Negative (-) | 1(reference) |  | 1(reference) |  |
| Positive (+) | 1.86 (1.78, 1.94) | <.001 | 1.34  (1.28, 1.40) | <.001 |
| Comorbidities |  |  |  |  |
| Hypertension | 3.87 (3.78, 3.97) | <.001 | 1.38  (1.34, 1.42) | <.001 |
| Diabetes mellitus | 2.07 (2.00, 2.13) | <.001 | 0.86  (0.83, 0.89) | <.001 |
| Dyslipidemia | 1.56 (1.52, 1.60) | <.001 | 0.93  (0.90, 0.96) | <.001 |
| Heart failure | 8.72  (8.37, 9.09) | <.001 | 2.51  (2.40, 2.63) | <.001 |
| Myocardial Infarction | 3.34  (2.95, 3.8) | <.001 | 0.98  (0.86, 1.11) | 0.701 |
| Valvular heart disease | 22.11  (20.80, 23.51) | <.001 | 6.07  (6.32, 7.21) | <.001 |
| Cardiomyopathy | 14.563  (12.83, 16.53) | <.001 | 2.79  (2.45, 3.18) | <.001 |
| Congenital heart disease | 9.02  (7.17, 11.34) | <.001 | 2.29  (1.81, 2.90) | <.001 |
| Hyperthyroidism | 2.09  (1.93, 2.26) | <.001 | 1.86  (1.72, 2.02) | <.001 |
| Charlson comorbidity index |  |  |  |  |
| 0 | 1(reference) |  | 1(reference) |  |
| 1 | 2.15 (2.09, 2.21) | <.001 | 1.13  (1.09, 1.16) | <.001 |
| ≥ 2 | 2.96 (2.85, 3.07) | <.001 | 1.15  (1.10, 1.20) | <.001 |
| eGFR, percentile |  |  |  |  |
| 1st (<67.51) | 2.45 (2.34, 2.56) | <.001 | 1.08  (1.03, 1.13) | 0.010 |
| 2nd (67.51-75.08) | 1.45 (1.38, 1.52) | <.001 | 1.05  (1.00, 1.10) | 0.034 |
| 3rd (75.08-80.69) | 1.04 (0.99, 1.09) | 0.170 | 1.05  (1.00, 1.11) | 0.056 |
| 4th (80.69-86.05) | 0.92 (0.87, 0.97) | 0.001 | 0.96  (0.91, 1.01) | 0.130 |
| 5th (86.05-90.49) | 1(reference) |  | 1(reference) |  |
| 6th (90.49-95.50) | 0.93 (0.89, 0.98) | 0.010 | 1.02  (0.97, 1.08) | 0.443 |
| 7th (95.50-100.55) | 0.75 (0.71, 0.79) | <.001 | 1.04  (0.98, 1.10) | 0.216 |
| 8th (100.55-105.63) | 0.46 (0.43, 0.49) | <.001 | 0.91  (0.85, 0.97) | 0.006 |
| 9th (105.63-113.41) | 0.32 (0.30, 0.34) | <.001 | 0.83  (0.77, 0.89) | <.001 |
| 10th (≥113.41) | 0.16 (0.14, 0.18) | <.001 | 0.77  (0.70, 0.85) | <.001 |

Multivariable model was adjusted with sex, age, income levels, smoking, alcohol consumption, regular physical activity, body mass index, waist circumference, proteinuria, hypertension, diabetes mellitus, dyslipidemia, heart failure, myocardial infarction, valvular heart disease, cardiomyopathy, congenital heart disease, hyperthyroidism, and Charlson comorbidity index.

HR, hazard ratio; CI, confidence interval; Q, quartile; eGFR, Estimated Glomerular Filtration Rate.

Supplementary Table 3. Association factors of renal function (ranges of estimated glomerular filtration) with the occurrence of atrial fibrillation

| Variable | Crude HR (95% CI) | p-value | Adjusted HR (95% CI) | p-value |
| --- | --- | --- | --- | --- |
| Sex |  |  |  |  |
| Male | 1(reference) |  | 1(reference) |  |
| Female | 0.67 (0.65, 0.68) | <.001 | 0.56 (0.54, 0.58) | <.001 |
| Age, years | 1.08 (1.08, 1.08) | <.001 | 1.07 (1.07, 1.07) | <.001 |
| Household income |  |  |  |  |
| Q1, lowest | 1(reference) |  | 1(reference) |  |
| Q2 | 0.92 (0.89, 0.95) | <.001 | 0.98 (0.95, 1.02) | 0.314 |
| Q3 | 1.19 (1.15, 1.23) | <.001 | 1.00 (0.96, 1.03) | 0.761 |
| Q4, highest | 1.58 (1.53, 1.64) | <.001 | 1.04 (1.00, 1.07) | 0.060 |
| Smoking status |  |  |  |  |
| Never | 1(reference) |  | 1(reference) |  |
| Former | 1.59 (1.54, 1.64) | <.001 | 1.06 (1.02, 1.10) | 0.002 |
| Current | 0.78 (0.75, 0.80) | <.001 | 0.91 (0.88, 0.95) | <.001 |
| Alcohol consumption (days/week) |  |  |  |  |
| None | 1(reference) |  | 1(reference) |  |
| 1-4 | 0.70 (0.68, 0.72) | <.001 | 1.10 (1.07, 1.13) | <.001 |
| ≥ 5 | 1.68 (1.61, 1.76) | <.001 | 1.25 (1.19, 1.32) | <.001 |
| Regular physical activity (days/week) |  |  |  |  |
| None | 1(reference) |  | 1(reference) |  |
| 1-4 | 0.76 (0.74, 0.78) | <.001 | 0.97 (0.94, 1.00) | 0.028 |
| ≥ 5 | 1.27 (1.21, 1.32) | <.001 | 1.01 (0.96, 1.05) | 0.798 |
| Body mass index (kg/m2) | 1.07 (1.07, 1.08) | <.001 | 1.04 (1.03, 1.04) | <.001 |
| Waist circumference (cm) | 1.01 (1.01, 1.01) | <.001 | 1.01 (1.01, 1.01) | <.001 |
| Proteinuria |  |  |  |  |
| Negative (-) | 1(reference) |  | 1(reference) |  |
| Positive (+) | 1.86 (1.78, 1.94) | <.001 | 1.34 (1.28, 1.40) | <.001 |
| Comorbidities |  |  |  |  |
| Hypertension | 3.87 (3.78, 3.97) | <.001 | 1.39 (1.35, 1.42) | <.001 |
| Diabetes mellitus | 2.07 (2.00, 2.13) | <.001 | 0.86 (0.83, 0.89) | <.001 |
| Dyslipidemia | 1.56 (1.52, 1.60) | <.001 | 0.93 (0.91, 0.95) | 0.005 |
| Heart failure | 8.72  (8.37, 9.09) | <.001 | 2.50  (2.39, 2.62) | <.001 |
| Myocardial Infarction | 3.34  (2.95, 3.8) | <.001 | 0.98  (0.86,1.11) | 0.696 |
| Valvular heart disease | 22.11  (20.80, 23.51) | <.001 | 6.76  (6.33, 7.22) | <.001 |
| Cardiomyopathy | 14.563  (12.83, 16.53) | <.001 | 2.80  (2.46, 3.19) | <.001 |
| Congenital heart disease | 9.02  (7.17, 11.34) | <.001 | 2.31  (1.82, 2.91) | <.001 |
| Hyperthyroidism | 2.09  (1.93, 2.26) | <.001 | 1.86  (1.72, 2.02) | <.001 |
| Charlson comorbidity index |  |  |  |  |
| 0 | 1(reference) |  | 1(reference) |  |
| 1 | 2.15 (2.09, 2.21) | <.001 | 1.13  (1.09, 1.16) | <.001 |
| ≥ 2 | 2.96 (2.85, 3.07) | <.001 | 1.15 (1.10, 1.20) | <.001 |
| eGFR, range |  |  |  |  |
| <30 | 4.23 (3.53, 5.08) | <.001 | 1.22 (1.01, 1.46) | 0.039 |
| 30-60 | 2.50 (2.41, 2.59) | <.001 | 1.02 (0.98, 1.06) | 0.308 |
| 60-90 | 1(reference) |  | 1(reference) |  |
| 90-120 | 0.46 (0.45, 0.48) | <.001 | 0.95 (0.92, 0.98) | <.001 |
| >120 | 0.13 (0.11, 0.15) | <.001 | 0.88 (0.78, 0.98) | 0.045 |

Multivariable model was adjusted with sex, age, income levels, smoking, alcohol consumption, regular physical activity, body mass index, waist circumference, proteinuria, hypertension, diabetes mellitus, dyslipidemia, heart failure, myocardial infarction, valvular heart disease, cardiomyopathy, congenital heart disease, hyperthyroidism, and Charlson comorbidity index.

HR, hazard ratio; CI, confidence interval; Q, quartile; eGFR, Estimated Glomerular Filtration Rate.

Supplementary Table 4. The subgroup analysis for the association of renal function (ranges of estimated glomerular filtration) with the occurrence of atrial fibrillation

| Variable | Adjusted HR (95% CI) | p-value for interaction effect |
| --- | --- | --- |
| Sex |  | 0.071 |
| Male |  |  |
| eGFR<30 | 1.12 (0.86, 1.46) |  |
| eGFR 30-60 | 0.98 (0.93, 1.04) |  |
| eGFR 60-90 | 1(reference) |  |
| eGFR 90-120 | 0.94 (0.91, 0.98) |  |
| eGFR >120 | 0.96 (0.81, 1.15) |  |
| Female |  |  |
| eGFR <30 | 1.29 (1.00, 1.68) |  |
| eGFR 30-60 | 1.06 (1.00, 1.12) |  |
| eGFR 60-90 | 1(reference) |  |
| eGFR 90-120 | 0.97 (0.92, 1.01) |  |
| eGFR >120 | 0.77 (0.59, 1.00) |  |
| Age, years |  | 0.008 |
| <50 |  |  |
| eGFR <30 | 1.23 (0.31, 4.96) |  |
| eGFR 30-60 | 1.12 (0.82, 1.55) |  |
| eGFR 60-90 | 1(reference) |  |
| eGFR 90-120 | 0.78 (0.73, 0.83) |  |
| eGFR >120 | 0.54 (0.45, 0.65) |  |
| ≥50 |  |  |
| eGFR <30 | 1.68 (1.40, 2.02) |  |
| eGFR 30-60 | 1.40 (1.35, 1.45) |  |
| eGFR 60-90 | 1(reference) |  |
| eGFR 90-120 | 0.72 (0.70, 0.75) |  |
| eGFR >120 | 0.84 (0.66, 1.06) |  |
| Body mass index (kg/m^2^) |  | 0.189 |
| <25 |  |  |
| eGFR <30 | 1.19 (0.93, 1.52) |  |
| eGFR 30-60 | 1.01 (0.96, 1.06) |  |
| eGFR 60-90 | 1(reference) |  |
| eGFR 90-120 | 0.92 (0.88, 0.95) |  |
| eGFR >120 | 0.80 (0.67, 0.96) |  |
| ≥25 |  |  |
| eGFR <30 | 1.23 (0.94, 1.62) |  |
| eGFR 30-60 | 1.03 (0.97, 1.09) |  |
| eGFR 60-90 | 1(reference) |  |
| eGFR 90-120 | 0.94 (0.90, 0.98) |  |
| eGFR >120 | 0.93 (0.74, 1.16) |  |
| Smoking status |  | 0.182 |
| Never |  |  |
| eGFR <30 | 1.21 (0.97, 1.52) |  |
| eGFR 30-60 | 1.02 (0.97, 1.06) |  |
| eGFR 60-90 | 1(reference) |  |
| eGFR 90-120 | 0.93 (0.89, 0.96) |  |
| eGFR >120 | 0.83 (0.68, 1.01) |  |
| Former |  |  |
| eGFR <30 | 1.03 (0.68, 1.55) |  |
| eGFR 30-60 | 0.97 (0.90, 1.06) |  |
| eGFR 60-90 | 1(reference) |  |
| eGFR 90-120 | 0.91 (0.85, 0.97) |  |
| eGFR >120 | 0.79 (0.55, 1.13) |  |
| Current |  |  |
| eGFR <30 | 1.63 (0.98, 2.73) |  |
| eGFR 30-60 | 1.04 (0.93, 1.17) |  |
| eGFR 60-90 | 1(reference) |  |
| eGFR 90-120 | 0.96 (0.90, 1.02) |  |
| eGFR >120 | 0.99 (0.76, 1.27) |  |
| Alcohol consumption (days/week) |  | 0.381 |
| None |  |  |
| eGFR <30 | 1.19 (0.97, 1.46) |  |
| eGFR 30-60 | 1.03 (0.98, 1.07) |  |
| eGFR 60-90 | 1(reference) |  |
| eGFR 90-120 | 0.93 (0.89, 0.97) |  |
| eGFR >120 | 0.84 (0.67, 1.05) |  |
| 1-4 |  |  |
| eGFR <30 | 1.62 (1.07, 2.44) |  |
| eGFR 30-60 | 0.97 (0.89, 1.05) |  |
| eGFR 60-90 | 1(reference) |  |
| eGFR 90-120 | 0.92 (0.88, 0.97) |  |
| eGFR >120 | 0.97 (0.79, 1.18) |  |
| ≥ 5 |  |  |
| eGFR <30 | 0.43 (0.06, 3.02) |  |
| eGFR 30-60 | 1.19 (1.02, 1.39) |  |
| eGFR 60-90 | 1(reference) |  |
| eGFR 90-120 | 0.94 (0.85, 1.04) |  |
| eGFR >120 | 0.69 (0.36, 1.33) |  |
| Regular physical activity (days/week) |  | 0.941 |
| None |  |  |
| eGFR <30 | 1.19 (0.96, 1.47) |  |
| eGFR 30-60 | 1.02 (0.98 1.07) |  |
| eGFR 60-90 | 1(reference) |  |
| eGFR 90-120 | 0.92 (0.88, 0.95) |  |
| eGFR >120 | 0.79 (0.66, 0.96) |  |
| 1-4 |  |  |
| eGFR <30 | 1.23 (0.76, 1.99) |  |
| eGFR 30-60 | 0.98 (0.90, 1.06) |  |
| eGFR 60-90 | 1(reference) |  |
| eGFR 90-120 | 0.96 (0.91, 1.01) |  |
| eGFR >120 | 1.03 (0.82, 1.31) |  |
| ≥ 5 |  |  |
| eGFR <30 | 1.51 (0.81, 2.04) |  |
| eGFR 30-60 | 1.04 (0.91, 1.20) |  |
| eGFR 60-90 | 1(reference) |  |
| eGFR 90-120 | 0.89 (0.80, 0.99) |  |
| eGFR >120 | 0.59 (0.03, 1.25) |  |
| Proteinuria |  | 0.350 |
| Negative (-) |  |  |
| eGFR <30 | 1.21 (0.95, 1.55) |  |
| eGFR 30-60 | 1.00 (0.96, 1.04) |  |
| eGFR 60-90 | 1(reference) |  |
| eGFR 90-120 | 0.93 (0.90, 0.96) |  |
| eGFR >120 | 0.85 (0.73, 0.98) |  |
| Positive (+) |  |  |
| eGFR <30 | 1.25 (0.94, 1.06) |  |
| eGFR 30-60 | 1.15 (1.03, 1.29) |  |
| eGFR 60-90 | 1(reference) |  |
| eGFR 90-120 | 0.94 (0.84, 1.05) |  |
| eGFR >120 | 0.98 (0.57, 1.68) |  |
| Comorbidities |  |  |
| Hypertension |  | 0.101 |
| No |  |  |
| eGFR <30 | 0.88 (0.46, 1.70) |  |
| eGFR 30-60 | 0.97 (0.90, 1.04) |  |
| eGFR 60-90 | 1(reference) |  |
| eGFR 90-120 | 0.95 (0.92, 0.99) |  |
| eGFR >120 | 0.94 (0.79, 1.12) |  |
| Yes |  |  |
| eGFR <30 | 1.32 (1.09, 1.60) |  |
| eGFR 30-60 | 1.08 (1.03, 1.13) |  |
| eGFR 60-90 | 1(reference) |  |
| eGFR 90-120 | 0.89 (0.86, 0.93) |  |
| eGFR >120 | 0.99 (0.77, 1.29) |  |
| Diabetes mellitus |  | 0.518 |
| No |  |  |
| eGFR <30 | 1.16 (0.91, 1.48) |  |
| eGFR 30-60 | 0.99 (0.95, 1.04) |  |
| eGFR 60-90 | 1(reference) |  |
| eGFR 90-120 | 0.93 (0.90, 0.96) |  |
| eGFR >120 | 0.86 (0.74, 1.01) |  |
| Yes |  |  |
| eGFR <30 | 1.36 (1.03, 1.80) |  |
| eGFR 30-60 | 1.10 (1.02, 1.19) |  |
| eGFR 60-90 | 1(reference) |  |
| eGFR 90-120 | 0.89 (0.83, 0.95) |  |
| eGFR >120 | 0.92 (0.60, 1.41) |  |
| Dyslipidemia |  | 0.283 |
| No |  |  |
| eGFR <30 | 1.08 (1.84, 1.38) |  |
| eGFR 30-60 | 0.98 (0.94, 1.03) |  |
| eGFR 60-90 | 1(reference) |  |
| eGFR 90-120 | 0.93 (0.90, 0.96) |  |
| eGFR >120 | 0.85 (0.72, 0.99) |  |
| Yes |  |  |
| eGFR <30 | 1.44 (1.09, 1.89) |  |
| eGFR 30-60 | 1.09 (1.02, 1.16) |  |
| eGFR 60-90 | 1(reference) |  |
| eGFR 90-120 | 0.92 (0.86, 0.97) |  |
| eGFR >120 | 0.97 (0.69, 1.38) |  |
| Heart Failure |  | 0.283 |
| No |  |  |
| eGFR <30 | 1.40 (1.14, 1.73) |  |
| eGFR 30-60 | 1.00 (0.96, 1.04) |  |
| eGFR 60-90 | 1(reference) |  |
| eGFR 90-120 | 0.94 (0.91, 0.96) |  |
| eGFR >120 | 0.89 (0.77, 1.03) |  |
| Yes |  |  |
| eGFR <30 | 1.05 (0.72, 1.54) |  |
| eGFR 30-60 | 1.17 (1.06, 1.29) |  |
| eGFR 60-90 | 1(reference) |  |
| eGFR 90-120 | 0.82 (0.73, 0.92) |  |
| eGFR >120 | 0.78 (0.37, 1.64) |  |
| Myocardial infarction |  | 0.558 |
| No |  |  |
| eGFR <30 | 1.23 (1.02, 1.48) |  |
| eGFR 30-60 | 1.01 (0.97, 1.05) |  |
| eGFR 60-90 | 1(reference) |  |
| eGFR 90-120 | 0.93 (0.90, 0.96) |  |
| eGFR >120 | 0.86 (0.74, 0.99) |  |
| Yes |  |  |
| eGFR <30 | 0.83 (0.20, 3.40) |  |
| eGFR 30-60 | 1.01 (0.74, 1.40) |  |
| eGFR 60-90 | 1(reference) |  |
| eGFR 90-120 | 0.86 (0.60, 1.23) |  |
| eGFR >120 | - |  |

Multivariable model was adjusted with sex, age, income levels, smoking, alcohol consumption, regular physical activity, body mass index, waist circumference, proteinuria, hypertension, diabetes mellitus, dyslipidemia, heart failure, myocardial infarction, valvular heart disease, cardiomyopathy, congenital heart disease, hyperthyroidism, and Charlson comorbidity index.

HR, hazard ratio; CI, confidence interval; eGFR, Estimated Glomerular Filtration Rate.

Supplementary Table 5. Association of renal function (MDRD method) with the occurrence of atrial fibrillation

|  | Adjusted HR (95% CI) | p-value |
| --- | --- | --- |
| eGFR (decile), mL/min/1.73 m^2^ |  |  |
| 1st (<67.51) | 1.17 (1.11, 1.24) | <.001 |
| 2nd (67.51-75.08) | 1.16 (1.10, 1.23) | <.001 |
| 3rd (75.08-80.69) | 1.08 (1.02, 1.15) | 0.009 |
| 4th (80.69-86.05) | 1.18 (1.11, 1.25) | <.001 |
| 5th (86.05-90.49) | 1(reference) |  |
| 6th (90.49-95.50) | 1.12 (1.06, 1.19) | <.001 |
| 7th (95.50-100.55) | 1.06 (0.99, 1.13) | 0.119 |
| 8th (100.55-105.63) | 1.04 (0.97, 1.11) | 0.279 |
| 9th (105.63-113.41) | 1.03 (0.96, 1.10) | 0.459 |
| 10th (≥113.41) | 0.90 (0.85, 0.95) | 0.029 |
| eGFR(range), mL/min/1.73 m^2^ |  |  |
| <30 | 1.19 (0.98, 1.45) | 0.076 |
| 30-60 | 1.03 (1.00, 1.07) | 0.067 |
| 60-90 | 1(reference) |  |
| 90-120 | 0.93 (0.90, 0.96) | <.001 |
| >120 | 0.87 (0.82, 0.93) | <.001 |

Multivariable model was adjusted with sex, age, income levels, smoking, alcohol consumption, regular physical activity, body mass index, waist circumference, proteinuria, hypertension, diabetes mellitus, dyslipidemia, heart failure, myocardial infarction, valvular heart disease, cardiomyopathy, congenital heart disease, hyperthyroidism, and Charlson comorbidity index.

HR, hazard ratio; CI, confidence interval; Q, quartile; eGFR, Estimated Glomerular Filtration Rate.

**Supplementary Methods**

The Chronic Kidney Disease Epidemiology Collaboration (CKD-EPI) equation was calculated as eGFR (mL/min/1.73m^2^):

eGFR=144×(SCr/0.7)^−0.329^× (0.993)^age^ (if female and SCr≤0.7 mg/dL), eGFR=144×(SCr/0.7)^−1.209^×(0.993)^age^ (if female and SCr>0.7 mg/dL), eGFR=144×(SCr/0.9)^−0.411^×(0.993)^age^ (if male and SCr≤0.9 mg/dL), and eGFR=144×(SCr>0.9)^−1.209^× (0.993)^age^ (if male and SCr>0.9 mg/dL).^1^

We identified hypertension according to the following criteria: 1) prescription of any antihypertensive medication with at least one claim of the diagnostic codes I10–15, 2) two or more claims of the diagnostic codes I10–15, 3) blood pressure ≥140/90 mmHg (systolic/diastolic), or 4) self-reported hypertension in the questionnaire. DM was defined as meeting one of the following criteria: 1) prescription of an antidiabetic agent with at least one claim of the diagnostic codes E11–14, 2) ≥2 claims of the diagnostic codes E11–14, 3) fasting serum glucose level≥7.0 mmol/L, or 4) self-reported DM in the questionnaire. Dyslipidemia was defined as satisfying one of the following criteria: 1) prescription of a dyslipidemia-related agent with at least one claim of the diagnostic code E78, 2) two or more claims of the diagnostic code E78, or 3) total cholesterol≥240 mg/dL. Charlson comorbidity conditions were collected using the Korean version of the Charlson comorbidity index.^2-8^ Heart failure was defined as at least one claim of the diagnostic codes I50, I50.x, I11.0, I13.1, I 13.0, I13.2.^9^ Myocardial infarction failure was defined as at least one claim of the diagnostic codes I21 and I22.^10^ Valvular heart disease was defined as at least one claim of the diagnostic codes A52.0, I05, I08, I09.1, I09.8, I34, I.39, Q23.0-23.3, Z95.2-95.4.^8^ Cardiomyopathy was defined as at least one claim of the diagnostic code I42.^11^ Congenital heart disease was defined as at least one claim of the diagnostic codes: ventricular septal defect (Q21.0, Q21.00, Q21.01, Q21.08, Q21.09), atrial septal defect (Q21.1, Q21.10, Q21.11, 21.18, Q21.19), atrioventricular septal defects (Q21.2), congenital malformation of the cardiac septum (Q21.9), patent ductus arteriosus (Q25.0), pulmonary artery stenosis (Q22.1, Q25.6), coarctation of aorta (Q25.1), pulmonary venous connection (Q26.2, Q26.3, Q26.4), congenital tricuspid stenosis (Q22.4, Q22.8, Q22.9), congenital stenosis of aortic valve (Q23.0), congenital insufficiency of aortic valve (Q23.1), congenital mitral stenosis (Q23.2, Q23.3), malformation of coronary vessels (Q24.5, Q24.8, Q24.9), stenosis or malformation of aorta (Q24.4, Q25.2, Q25.3, Q25.4, Q25.8, Q25.9), Tetralogy of Fallot (Q21.3), Ebstein anomaly (Q22.5), transposition of great arteries (Q20.3, Q20.5), Eisenmenger syndrome (Q21.81, I21.81), double outlet right ventricle (Q20.1), and single ventricle (Q20.4).^12^ Hyperthyroidism was defined as at least one claim of the diagnostic code E05.^13^

**References**

1. Tent H, Waanders F, Krikken JA, Heerspink HJ, Stevens LA, Laverman GD, et al. Performance of MDRD study and CKD-EPI equations for long-term follow-up of nondiabetic patients with chronic kidney disease. Nephrol Dial Transplant. 2012;27 Suppl 3:iii89-95.

2. Song TJ, Kim JW, Kim J. Oral health and changes in lipid profile: A nationwide cohort study. J Clin Periodontol. 2020;47(12):1437-45.

3. Woo HG, Chang Y, Lee JS, Song TJ. Association of Tooth Loss with New-Onset Parkinson's Disease: A Nationwide Population-Based Cohort Study. Parkinsons Dis. 2020;2020:4760512.

4. Chang Y, Woo HG, Lee JS, Song TJ. Better oral hygiene is associated with lower risk of stroke. J Periodontol. 2021;92(1):87-94.

5. Lee K, Lee JS, Kim J, Lee H, Chang Y, Woo HG, et al. Oral health and gastrointestinal cancer: A nationwide cohort study. J Clin Periodontol. 2020;47(7):796-808.

6. Kim J, Kim HJ, Jeon J, Song TJ. Association between oral health and cardiovascular outcomes in patients with hypertension: a nationwide cohort study. J Hypertens. 2022;40(2):374-81.

7. Song TJ, Chang Y, Jeon J, Kim J. Oral health and longitudinal changes in fasting glucose levels: A nationwide cohort study. PLoS One. 2021;16(6):e0253769.

8. Kim, K.H. Comorbidity Adjustment in Health Insurance Claim Database. Health Policy and Management. 2016;26(1):71-78

9. Park JJ, Lee CJ, Park SJ, Choi JO, Choi S, Park SM, et al. Heart Failure Statistics in Korea, 2020: A Report from the Korean Society of Heart Failure. Int J Heart Fail. 2021;3(4):224-36.

10. Choi EK. Cardiovascular Research Using the Korean National Health Information Database. Korean Circ J. 2020;50(9):754-72.

11. Lee JH, Lim NK, Cho MC, Park HY. Epidemiology of Heart Failure in Korea: Present and Future. Korean Circ J. 2016;46(5):658-64.

12. Jang SY, Seo SR, Moon JR, Cho EJ, Kim E, Chang SA, et al. Prevalence and mortality of congenital heart disease in Korean adults. Medicine (Baltimore). 2018;97(27):e11348.

13. Lee SR, Ahn HJ, Choi EK, Lee SW, Han KD, Oh S, et al. Improved prognosis with integrated care management including early rhythm control and healthy lifestyle modification in patients with concurrent atrial fibrillation and diabetes mellitus: a nationwide cohort study. Cardiovasc Diabetol. 2023;22(1):18
